# First record of the genus *Schistomitra* Butler, 1881 (Lepidoptera, Epicopeiidae) from China, with the description of a new species

**DOI:** 10.3897/zookeys.878.35364

**Published:** 2019-10-07

**Authors:** Si-Yao Huang, Yuan Zhang, Min Wang, Xiao-Ling Fan

**Affiliations:** 1 Department of Entomology, College of Agriculture, South China Agricultural University, Guangzhou 510642, Guangdong, China South China Agricultural University Guangzhou China; 2 School of Life Sciences, Sun Yat-Sen University, Guangzhou 510006, China Sun Yat-Sen University Guangzhou China

**Keywords:** East Asia, Geometroidea, host plant, oriental swallowtail moth, *Stewartia*, taxonomy

## Abstract

The epicopeiid moth genus *Schistomitra* Butler, 1881 is reported outside Japan for the first time, with a new species, *Schistomitra joelmineti* Huang & Wang, **sp. nov.**, described from the southern part of Shaanxi and Gansu Province in China. Photographs of adults and genitalia are provided, and the distribution pattern of the genus is discussed.

## Introduction

Epicopeiidae Swinhoe, 1892, commonly known as oriental swallowtail moths, belongs to the superfamily Geometroidea within Macroheterocera*sensu*Mitter et al. (2017). Hitherto this small family is comprised of 10 genera and less than 30 species restricted in the Asian Palaearctic and Oriental regions. The family can be distinguished by having a head without ocelli, forewing without an areole and in the forewing venation – vein R_5_ usually stalked with vein M_1_ (Minet 2003). Members of the family nearly all come out at day with few exceptions, and are usually mimickers of some diurnal lepidopterous families such as Papilionidae, Pieridae, Riodinidae and Zygaenidae. Immature stages are only known in the genera *Epicopeia* Westwood, 1841, *Psychostrophia*Butler, 1877 and *Schistomitra* Butler, 1881, and their larva and pupa are covered by a waxy substance on the surface (Janet and Wytsman 1903, Sugi 1972, Nakamura 2006).

The genus *Schistomitra* was erected by Butler in 1881 based on its type species, *S. funeralis* Butler, 1881. It is characterized by the following two characters: labial palpi entirely blackish brown and gnathos represented by a pair of short and sclerotized arms which are dentate dorsally (Minet 2003). Externally *Schistomitra* is unique within Epicopeiidae by imagoes having an extensive pale yellowish ground color which is divided into patches of different sizes and shapes by blackish patterns on the upper sides of wings. Adults of *S. funeralis* are diurnal, occurring from late spring to early summer, with numbers usually peaking between mid-May and mid-June. They can be found sucking nectar from flowers and water on damp ground. The larva, covered externally by waxy matter commonly found in other Epicopeiidae larvae, feeds on *Stewartia pseudocamellia* (Korean stewartia, Japanese stewartia, or deciduous camellia). It overwinters as a pupa (Sugi 1972).

Inoue (1982) established Schistomitrinae based on the genus *Schistomitra*, which contained two genera from Japan, viz. *Schistomitra* Butler, 1881 and *Psychostrophia* Butler, 1877, as a new subfamily under Epiplemidae (now the Epipleminae under Uraniidae). Minet (2003) synonymized Schistomitrinae with Epicopeiidae because in the analysis based on morphological characters proposed in the same paper, *Schistomitra* was found to be the sister group of the clade (*Nossa* Kirby, 1892 + *Epicopeia* Westwood, 1841); thus, there was no need to maintain this subfamily. In the molecular phylogenetic tree proposed in Wei and Yen (2017), *Schistomitra* was found to be the sister group of either the clade (*Chatamla* Moore, 1881 + *Mimaporia* Wei & Yen, 2017) or the clade (*Chatamla* Moore, 1881 + *Mimaporia* Wei & Yen, 2017 + *Parabraxas* Leech, 1897). Hence, the precise relationships between these genera is still unclear and needs further investigation.

For a long time, *Schistomitra* was regarded as an endemic Japanese genus only found in Honshu, Shikoku, and Kyushu (Sugi 1972, Owada 2011, Association of Aso area world agriculture inheritance promotion 2014). Unexpectedly, recently we received some interesting epicopeiid moth material which was collected from southern Shaanxi and Gansu from 2007 to 2017, and they shared many external similarities with the genus *Schistomitra*. After examining the genitalia and comparing them with those of *Schistomitra funeralis*, all the results lead to a conclusion that these materials belong to an undescribed species of the genus *Schistomitra* and it is described here.

## Materials and methods

Specimens examined in this study were all collected during the day with an insect net and are deposited in the collection of South China Agricultural University (**SCAU**), Guangzhou, PR China and the Bavarian State Collection of Zoology (Zoologische Staatssammlung München), Munich, Germany (**ZSM**). All adult photographs in the laboratory were taken with a Nikon CoolPix S7000 camera, the adults in wild and habitat photos were taken with a Canon EOS 80D camera by collector Mr Wen-hao Sun. Abdomens were removed and macerated in 10% NaOH for examination of male and female genitalia. Photographs of genitalia were all taken under a Keyence VHX-5000 digital microscope, and were all processed by Adobe Photoshop CS5 software. Terminology of adult morphology and genitalia follows Klots (1970), Kristensen (2003), Minet (2003), and Wei and Yen (2017).

## Taxonomy

### Genus Schistomitra

Taxon classificationAnimaliaLepidopteraEpicopeiidae

Butler, 1881

FA872957-1E38-5BF4-B571-3E8BB3E2BD57

Schistomitra Butler, 1881: 3.

#### Type species.

*Schistomitra funeralis* Butler, 1881 [Type locality: Fusiyama, Nikko (Honshu, Japan)].

### Schistomitra joelmineti

Taxon classificationAnimaliaLepidopteraEpicopeiidae

Huang & Wang sp. nov.

D8F3530D-D355-59B1-A282-2F8844C35A29

http://zoobank.org/8063CEFF-BE04-4007-A627-45C0EEEE187E

Figs 1–5 , 9–14 , 18–19 , 21 , 22

#### Type material.

***Holotype:***male, altitude 800–1000 m, 22.IV.2017, Chengguan Town, Ningshan County, Shaanxi Province, PR China, leg. Di Lu & Wen-hao Sun (SCAU). ***Paratype:*** 1 female, same data as holotype; 1 female, 26.V.2007, Houzhenzi Town, Zhouzhi County, Xi’an City, Shaanxi Province, PR China, leg. Hong-liang Shi (SCAU); 1 male, 1 female, altitude 1300–1500 m, 3.V.2009, Xunyangba Town, Ningshan County, Shaanxi Province, PR China, leg. Yu-fei Li (SCAU); 1 female, same locality and collector, but altitude 1500–1900 m, 8.VI.2014 (SCAU); 2 female, altitude 1500 m, 30.V.2017, Jialingjiang Head Water, Baoji City, Shaanxi Province, PR China, leg. Shu-qin Ji (SCAU); 1 male, altitude 1800 m, 29.IV.2017, Qinghe Forestry Farm, Kang County, Longnan City, Gansu Province, leg. Hao Huang (SCAU), 1 male, same locality and collector, but 1.V.2017 (SCAU); 1 female, same locality and collector, but altitude 1300 m, 3.VI.2017 (SCAU); 3 males, 1 female, altitude 1400 m, 4.VI.1991, Fengxiang County, Mts. Qin Ling, S. Shaanxi, PR China, leg. G.C. Bozano (ZSM).

#### Diagnosis.

*Schistomitra joelmineti* sp. nov. is characterized and distinguished from *S. funeralis* (Figs 6–8, 15–17, 20) by the following characters:

**Figures 1–8.**
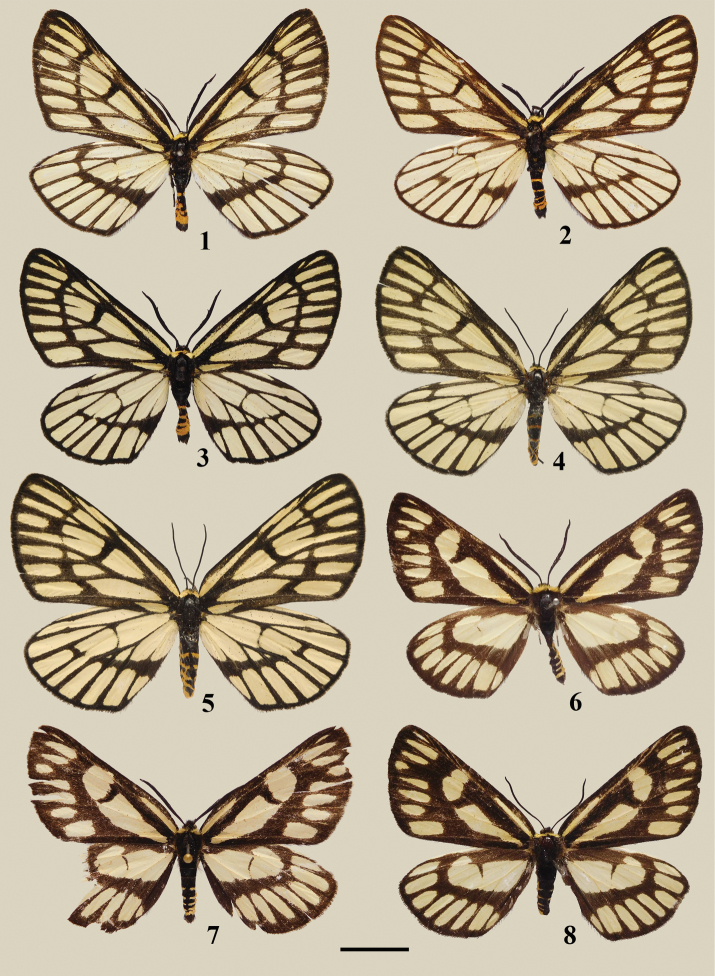
Adults of *Schistomitra* spp. **1–3, 6–7** male **4, 5, 8** female **1–5***Schistomitra joelmineti* sp. nov. **6–8***Schistomitra funeralis.* Scale bar: 1 cm.

**Figures 9–17.**
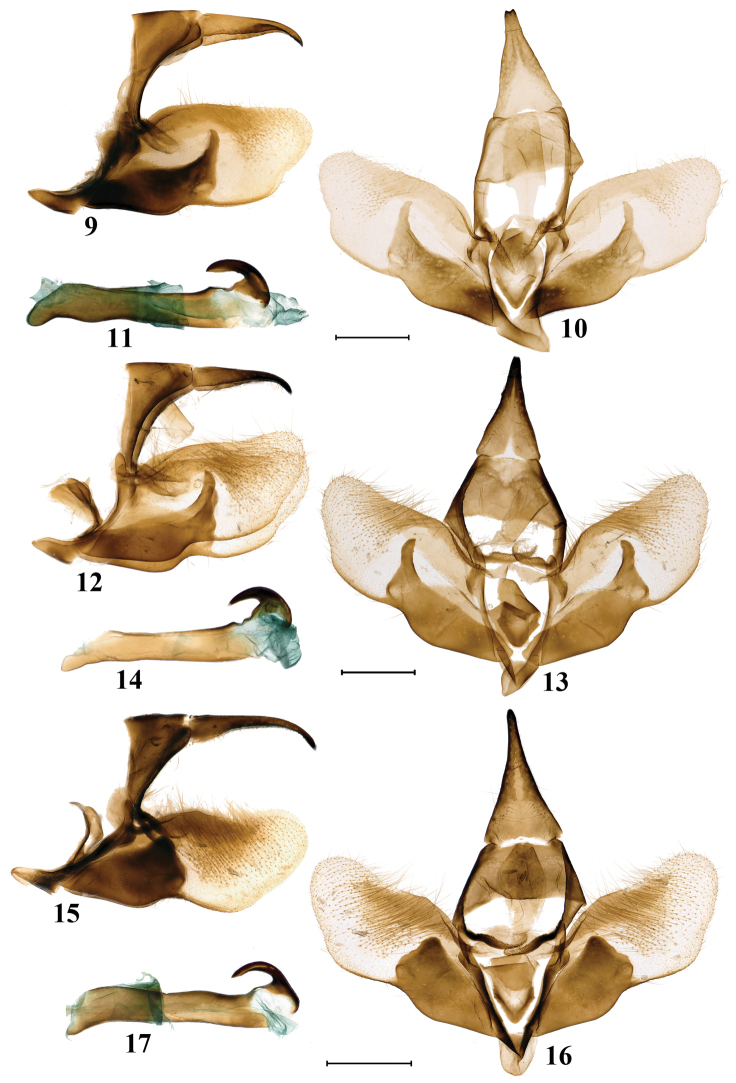
Male genitalia of *Schistomitra* spp **9–11** holotype of *Schistomitra joelmineti* sp. nov. **12–14** paratype of *Schistomitra joelmineti* sp. nov., from individual in Fig. 2**15–17***Schistomitra funeralis***9, 12, 15** genitalia capsule lateral view **10, 13, 16** genitalia capsule ventral view **11, 14, 17** aedeagus lateral view. Scale bars: 1 mm.

**Figures 18–20.**
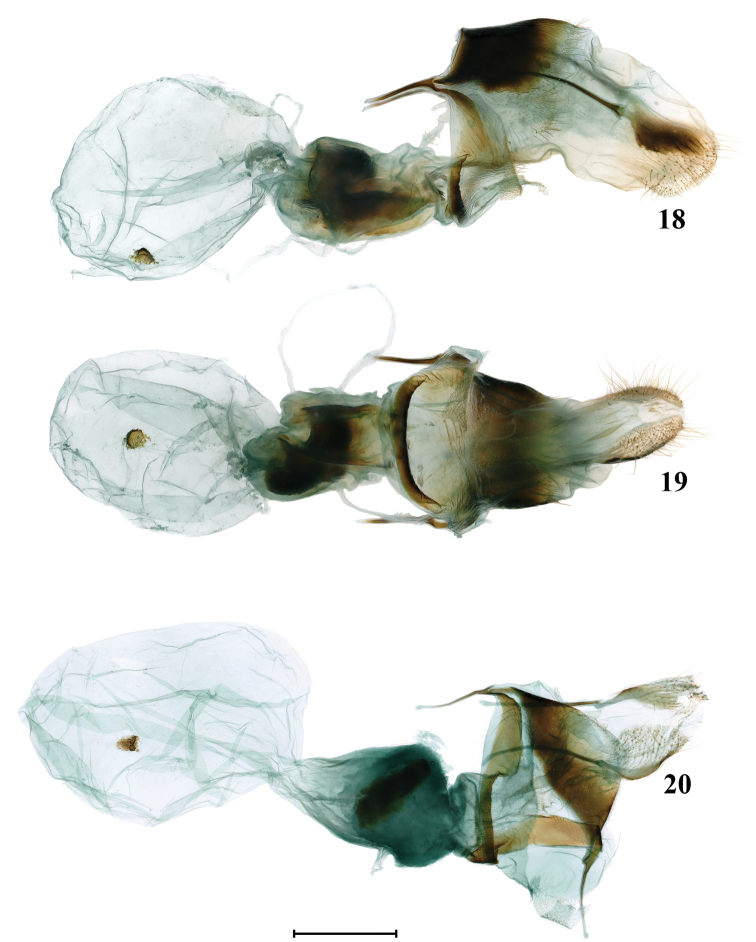
Female genitalia of *Schistomitra* spp. **18***Schistomitra joelmineti* sp. nov. lateral view **19***ditto*, ventral view **20***Schistomitra funeralis* ventral view. Scale bar: 1 mm.

1) the size is larger in both sexes, length of forewing 26–28 mm vs. 25–27 mm in males, 27–30 mm vs. 25 mm in females;

2) the forewing has the discoidal cell totally encircled by darkened veins, while the lower portion of discoidal cell remains pale yellow like its ground color in *S. funeralis*;

3) the blackish postmedian band on forewing upper side is narrower compared to the much wider band in *S. funeralis*;

4) the hind wing upper side has a much reduced blackish pattern in cell Rs and bases of cell 1A+2A and 3A, whereas the blackish pattern is better developed in all these cells in *S. funeralis*;

5) in the male genitalia the uncus is shorter with its tip nearly flat or slightly concave in the middle, while uncus is longer with its tip rounded in *S. funeralis*;

6) the sacculus is longer, and the apex of praesacculus forms a long, sharp, blade-like process pointing dorsally, while in *S. funeralis* the sacculus is shorter, with the apex only forming a short and rounded bulge;

7) the aedeagus is slightly thicker and longer, with the distal shaft more robust and the coecum larger, while the aedeagus is narrower and shorter, with distal shaft slenderer and coecum smaller in *S. funeralis*;

8) in the female genitalia, ductus bursae is more sclerotized, corpus bursae is smaller with a rounded signum, while in those of *S. funeralis* the ductus bursae is more membranous, the corpus bursae is larger, with the signum being elliptical.

#### Description.

***Male*** (Figs 1–3). Length of forewing 26–28 mm. Head black, frons wide, covered with long blackish hairy scales; vertex covered with long blackish brown hair; compound eye black and large; antenna black, bipectinate. Thorax black; patagia covered with pale yellow hairy scale; tegula black, cephalic part covered with pale yellow hairy scales; abdomen covered with dense black scales dorsally and ventrally with bright yellowish orange rings presenting at intersegmental membranes between each segment of abdomen. Forewing upper side ground color pale yellow, veins all broadly suffused with blackish scales except the base of vein M_2_, cilia black. Costa black from base to apex; postmedian band black, running from costal zone to dorsum and extending basally in cell 1A+2A; the pale yellow ground pattern from median to marginal zone is divided by darkened veins and postmedian band into smaller patches of various shape and size. Hindwing upper side ground color pale yellow, cilia black. All veins suffused with blackish scales. A black postmedian band extending from costa to dorsum, varying from fully developed to obsolete.

***Female*** (Figs 4, 5). Length of forewing 27–30 mm. Head, thorax and abdomen same as in male, antenna filiform. Forewing broader, termen more rounded, pattern nearly same as in male, only the blackish postmedian band on forewing not continuous. Hindwing slightly broader, other characters same as in male.

#### Genitalia.

***Male*** (Figs 9–14). Uncus moderately long and triangular, with its tip nearly flat or shallowly concave at the middle. Tegumen broad and short, trapezoid. Subscaphium slightly sclerotized. Gnathos consisted of the two sclerotized arms which connected by membrane medially and dorsally covered by many teeth of different size. Costula at the base of costa, consists of two sclerotized triangular processes connected together by a membrane. Juxta V-shaped, with each lobe extending upwards and ending with a large triangular-like sclerotized plate. Saccus broad and stout, semielliptical. Valva broad and short, concave at ventral margin with inner surface setose at apex. Costa moderately long, strongly sclerotized. Sacculus nearly the same length of costa, strongly sclerotized and slightly broadening at middle portion. Praesacculus short and strongly sclerotized, its apex ending with a long and sharp blade-like process directed dorsally, which slightly varying in width. Aedeagus long and thick, distal shaft strongly sclerotized and horn-shaped, bending at the middle and basally connecting to the dorsal wall of the aedeagus by a sclerotized plate, coecum presents, moderately developed.

***Female*** (Figs 18, 19). Papillae anales slightly sclerotized, rectangular in lateral view, with tip flat. Apophyses posteriores and anteriores sclerotized and slender; and the former are nearly twice the length of the latter. Antrum membranous and broad. Ostium bursae nearly the same width as antrum. Lamella antevaginalis broad, mostly membranous and anteriorly edging with a strongly sclerotized bar. Lamella postvaginalis obsolete and very slightly sclerotized, elliptical in ventral view. Ductus bursae short and broad, strongly sclerotized. Ductus seminalis very long, arising from ductus bursae. Corpus bursae membranous, large and oval shape. A sclerotized rounded signum presenting at anterior zone of corpus bursae, with numerous spinules on the surface.

#### Distribution.

Currently this species is restricted to the southern part of Shaanxi Province and Gansu Province.

#### Etymology.

The specific name *joelmineti* is named in honor of Prof. Joël Minet (Paris, France) who contributed greatly to the study of the family Epicopeiidae and kindly provided the first author with valuable literature when he began studying Epicopeiidae.

#### Biology.

**Figures 21–23.**
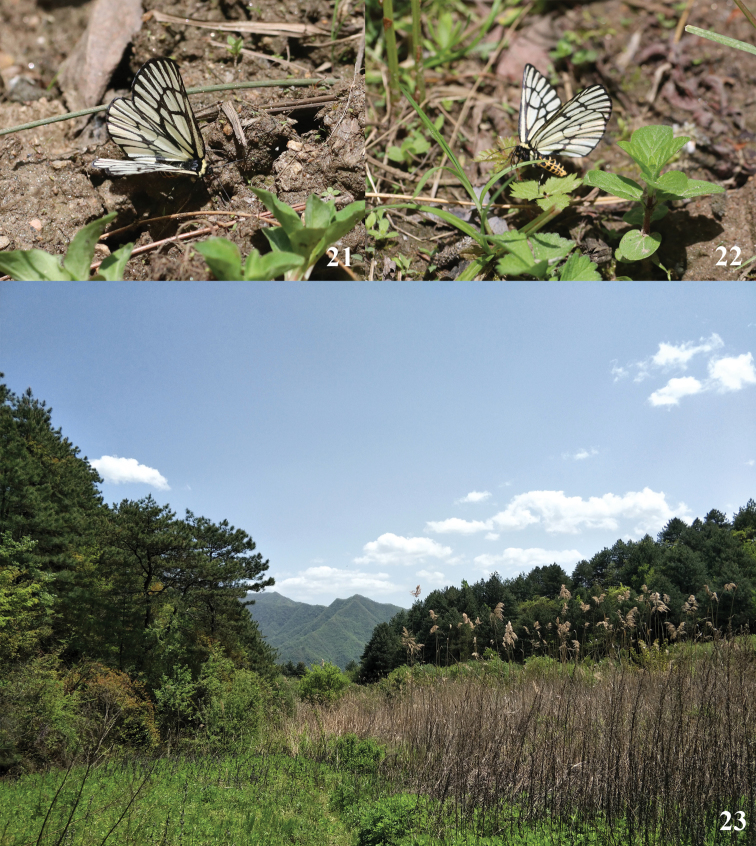
*Schistomitra joelmineti* sp. nov. living adult and habitat **21** sucking on damp ground **22** resting on leaves **23** habitat of *Schistomitra joelmineti* sp. nov. in Chengguan Town, Ningshan County.

This species is univoltine, occurring from late April to early June. Adults are usually found sucking nutrients and water on damp ground (Fig. 21) or resting on leaves (Fig. 22) near the edge of the forest (Fig. 23) at altitude between 800 to 1800 m.

## Discussion

Although the external features of adults of *Schistomitra joelmineti* are variable among individuals to some extent, their genitalia are only slightly different in the tip of the uncus, length of the aedeagus, and width of the pointed process in the praesacculus, while they differ greatly from those of *S. funeralis*. Thus, we regard all these individuals from China as a single species.

**Figure 24.**
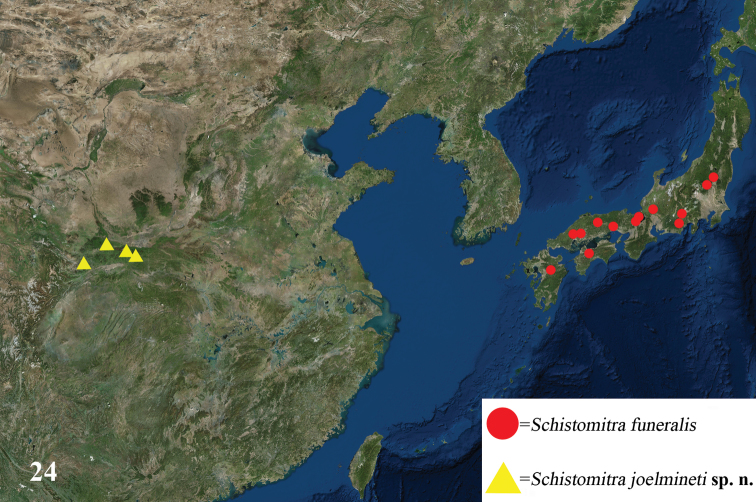
Distribution map of the genus *Schistomitra*. Records of distribution are taken from Sugi (1972), Association of Aso area world agriculture inheritance promotion (2014), and the present study.

The genus *Schistomitra* currently shows a disjunct distribution with the discovery of *Schistomitra joelmineti*, with one species found in the mountainous region around Mt. Qinling in Shaanxi Provinceand Gansu Province in China and the other in Honshu, Shikoku, and Kyushu in Japan (Fig. 24). The disjunct distribution might be caused by the distribution pattern of their host plants. The host plant genus *Stewartia* is widely distributed in Honshu, Shikoku, and Kyushu in Japan, the southern part of the Korean Peninsula, as well as in the vast area between Mt. Qinling and Mt. Nanling in China (Li 2011), which could explain the absence of this moth genus in northeastern, northern China and the northern part of the Korean Peninsula. However, it is still a mystery as to why this genus is not found in the southern part of the Korean Peninsula, but this will only be solved with careful and intensive collecting in the suitable habitats, to determine its biogeography. In addition, *S. funeralis* tends to be distributed in mountainous areas with a rather dry climate in Japan, where annual precipitation is no more than 1800 mm (Sugi 1972). Similarly, the mountainous regions in southern Shaanxi and Gansu are also rather cool and dry with an annual precipitation of no more than 1300 mm, similar to the habitats of *S. funeralis* in Japan. Thus, the narrow distribution of *Schistomitra* in southern Shaanxi and Gansu may relate to its intolerance of high temperatures or humid climates; in other words, the hot and humid climate of other regions may have played an important role in preventing the extension of this genus to southern and southeastern China.

Speciation in *Schistomitra* may mostly be the result of isolation mechanisms mentioned above. In addition, the utilization and distribution of different host plants may also contribute to it to some extent. In the phylogenetic tree of *Stewartia* proposed by Li (2011) based on morphological characters, *Stewartia pseudocamellia*, the host plant of *Schistomitra funeralis*, was not recovered in the same clade as the only species of *Stewartia* found in southern Shaanxi, *Stewartia shensiensis* (which might be the candidate host plant of *Schistomitra joelmineti*). In Lepidoptera, a similar case exists in the lycaenid butterfly genus *Sibataniozephyrus* Inomata, 1986, which is widespread in Japan, Taiwan, and the southern, southwestern and central part of mainland China, and diverges into three species utilizing different species of *Fagus* present in each area (Koiwaya 2007). Although Koiwaya suggested that *Sibataniozephyrus lijinae* Hsu, 1995 from mainland China might utilize the same *Fagus* species as *S. kuafui* Hsu & Lin, 1994 from Taiwan, there is still no evidence to support this idea.

## Supplementary Material

XML Treatment for Schistomitra

XML Treatment for Schistomitra joelmineti
